# Electroencephalographic Functional Connectivity Patterns in Children With Tourette Syndrome and Attention-Deficit/Hyperactivity Disorder

**DOI:** 10.1016/j.pediatrneurol.2025.08.001

**Published:** 2025-08-08

**Authors:** Simon Morand-Beaulieu, Julia Zhong, Karim Ibrahim, Michael J. Crowley, Heidi Grantz, James F. Leckman, Denis G. Sukhodolsky

**Affiliations:** a Department of Psychology, McGill University, Montréal, QC, Canada; b Child Study Center, Yale University School of Medicine, New Haven, Connecticut

**Keywords:** Tourette syndrome, Attention-deficit/hyperactivity disorder, Electroencephalography, Functional connectivity, Neural oscillations

## Abstract

**Background:**

Tourette syndrome (TS) and attention-deficit/hyperactivity disorder (ADHD) often co-occur and are linked to emotional and behavioral difficulties. However, their shared and distinct neural underpinnings, particularly in terms of functional connectivity, remain unclear. Here, we assessed how functional connectivity differs across TS and ADHD as well as its association with emotional and behavioral difficulties.

**Methods:**

Resting-state electroencephalography (EEG) was recorded from 137 children with TS (n = 51), ADHD (n = 24), or TS + ADHD (n = 29) or from typically developing control subjects (n = 33). Functional connectivity was computed from source-reconstructed EEG data in five frequency bands (delta, theta, alpha, beta, and gamma). Behavioral and emotional problems were assessed with the Child Behavior Checklist.

**Results:**

Both TS and ADHD were independently associated with reduced functional connectivity across different brain regions, with no interaction effect. However, externalizing problems showed a TS by ADHD interaction across three frequency bands, such that distinct patterns of functional connectivity were associated with externalizing problems in children with TS + ADHD, relative to those with either TS or ADHD.

**Conclusions:**

Although TS and ADHD are associated with decreased functional connectivity in different networks, their effects appear additive rather than interactive at the neural level. However, interactions emerged when examining behavioral problems, suggesting that although TS and ADHD contribute independently to brain connectivity disruptions, their combined impact may uniquely influence emotional and behavioral functioning. This fact highlights the need to consider both shared and disorder-specific mechanisms when studying TS and ADHD.

## Introduction

Tourette syndrome (TS) and attention-deficit/hyperactivity disorder (ADHD) frequently co-occur. In community samples, ADHD is present in 17%-52% of children with TS,^1,2^ whereas clinical samples report rates over 60%.^3^ Relative to children with TS only, children with both TS and ADHD show greater cognitive impairments^4,5^ as well as more psychosocial, emotional, and behavioral difficulties.^5–8^ Children with TS + ADHD also experience greater irritability,^9^ disruptive behaviors,^10^ and explosive outbursts.^11^ However, the extent to which the neurobiological underpinnings of TS and ADHD overlap remains unclear. It is also unknown whether psychosocial, emotional, and behavioral difficulties are linked with similar brain correlates in both conditions. Several models have been proposed to account for the common TS and ADHD comorbidity.^12–15^ Among those, the additive model suggests that TS and ADHD are two separate disorders whose effects are combined in individuals with TS + ADHD. Another, the interactive model, suggests that the TS + ADHD combination could represent a distinct nosological entity with symptoms including tics and inattention/hyperactivity. Finally, the phenotype model proposes that TS + ADHD could represent a phenotypical subgroup of either TS or ADHD.

To disentangle TS and ADHD, researchers often use a 2 × 2 factorial design with four groups: children with TS, ADHD, TS + ADHD, and typically developing children. Studies investigating the biological and neurofunctional correlates of TS and ADHD through genetics, structural and functional brain imaging, neurochemistry, and neurophysiology tend to support the additive model.^14,16^ However, some electrophysiological studies indicate subadditive^17^ or interactive effects,^18–20^ especially during tasks involving enhanced cognitive demands. Similarly, neurocognitive studies have yielded mixed evidence, with some reporting additive effects (i.e., worse performance in TS + ADHD relative to TS only)^21–23^ and others reporting subadditive effects (i.e., better performance in TS + ADHD relative to ADHD only).^24,25^ To reconcile these discrepancies, Banaschewski et al.^13^ proposed a stepwise model: the additive model may hold true for basic neurobiological aspects, but TS and ADHD could interact for higher-order cognitive processes. Nevertheless, further research is needed to clarify the complexities of their co-occurrence.

In recent years, functional and effective connectivity has been used to assess the underlying mechanisms of co-occurrence of TS and ADHD. These techniques address patterns of co-activation of different brain regions and may inform differences in neural communication across different disorders or conditions. Recent research using such approaches suggests mostly distinct neural communication patterns in TS and ADHD, with limited evidence for interactive effects when both conditions co-occur.^24,26^ However, methodological limitations remain. For instance, some research using electroencephalography (EEG) focused on broadband spectral analyses, thus potentially overlooking connectivity patterns confined to specific frequency bands. This is an important consideration given that different oscillatory frequencies may support distinct cognitive functions.^27^ The absence of factorial designs in some work also limits the ability to test additive versus interactive effects directly.

Beyond categorical diagnoses, functional connectivity has also been assessed in relation to symptom severity. For example, positive associations between tic severity and functional connectivity have been reported for the default mode and frontoparietal networks,^26,28^ whereas negative associations have been reported in the sensorimotor areas.^28,29^ However, associations with ADHD symptom severity remain inconsistent. Openneer et al.^26^ found no link between functional connectivity and ADHD symptom severity, whereas Jurgiel et al.^24^ reported that reduced connectivity in ADHD was associated with greater emotional and behavioral problems, although they did not assess whether these associations varied across diagnoses of TS and/or ADHD. These mixed findings suggest that the relationship between functional connectivity and symptoms frequently associated with TS and ADHD remains poorly understood.

The neural mechanisms underlying the co-occurrence of TS and ADHD remain unclear, with evidence supporting both additive and interactive effects at different levels of investigation. Functional connectivity offers a valuable insight for identifying shared or distinct patterns of neural synchronization in these conditions. Emerging models suggest that additive effects may occur at basic neurobiological levels, whereas interactive effects may emerge in more complex behavioral domains.^14^ Assessing functional connectivity alongside measures of symptom severity and behavioral and emotional functioning may help understand how additive and interactive effects of TS and ADHD manifest across different levels of analysis. Therefore, the first aim of this study was to assess how TS and ADHD separately and jointly impact functional brain connectivity across several frequency bands, among children aged seven to 14 years. This age range was selected to focus on the developmental period when tics are the most severe. Based on the literature, we expected that the ADHD factor, rather than TS, would be responsible for most differences in functional connectivity and that those differences would mostly involve connections between posterior cortical regions. The second aim of our study was to assess whether functional connectivity is associated with the severity of disruptive behavior, externalizing and internalizing problems, ADHD symptoms, and tic severity. We notably wanted to see if those associations differ across children with TS and/or ADHD. As those analyses were exploratory, we did not formulate specific hypotheses for this aim.

## Methods

### Participants

We recruited 138 children to participate in this study. These children were recruited through the Yale TS/obsessive-compulsive disorder specialty clinic and the Tourette Association of America’s Connecticut chapter. Inclusion criteria were (1) individuals ranging in age from seven to 14 years and (2) primary diagnosis of either TS or ADHD for children in the clinical groups, and the absence of any psychiatric disorder for the typically developing control group. Exclusion criteria were a history of (1) neurological illness, seizures, or head trauma accompanied by loss of consciousness; (2) intellectual disability indicated by an intelligence quotient below 70; (3) a diagnosis of autism spectrum disorder; and/or (4) a severe psychiatric disorder that could impede participation in the study. Medication intake was not an exclusion criterion. Children were assigned to one of the four groups following clinical assessment (see below). The clinical assessment revealed symptoms of autism spectrum disorder in one child initially recruited as a control participant, who was excluded from this study. Therefore, 137 children were included in the final sample. These children had TS (n = 51), ADHD (n = 24), or co-occurring TS and ADHD (n = 29) or were typically developing control subjects (n = 33). Clinical and demographic data for each group are presented in Table 1. This study was conducted according to the Declaration of Helsinki and was approved by Yale Institutional Review Board (#0411027198). Parents and children participating in the study respectively provided consent and assent before their participation.

### Procedures

#### Clinical assessment

Children were initially recruited based on a prior clinical diagnosis (or the lack thereof, for typically developing children), but final group assignment was determined following clinical assessment. A trained clinician administered a semistructured clinical interview (Kiddie Schedule for Affective Disorders and Schizophrenia)^30^ to comprehensively evaluate the presence of diagnoses of TS, ADHD, and any co-occurring disorders. The severity of ADHD, disruptive behaviors, and emotional and behavioral problems was assessed with parent-report measures. Severity of inattention and hyperactivity symptoms was assessed with the 18-item Swanson, Nolan, and Pelham Questionnaire (SNAP-IV).^31^ This questionnaire rates the severity of the nine inattentive and the nine hyperactive symptoms of ADHD according to the *Diagnostic and Statistical Manual of Mental Disorders, Fourth Edition, Text Revision* (DSM-IV-TR). Disruptive behaviors were assessed with the eight-item Disruptive Behavior Rating Scale (DBRS).^32^ Behavioral and emotional problems were assessed with Child Behavior Checklist (CBCL). In all children, best estimate DSM-IV-TR diagnoses of TS, ADHD, and concomitant disorders were determined from information gathered by clinical interviews and parent ratings of symptom severity.^33^

In both groups of children with TS, tic severity was assessed by expert clinicians using the total tic score of the Yale Global Tic Severity Scale (YGTSS).^34^ With the YGTSS, motor and phonic tics are assessed on a six-point scale (0–5) according to five dimensions: number, frequency, intensity, complexity, and interference. These dimensions are summed to yield motor and phonic tic severity scores, each ranging from 0 to 25. The total tic score consists of the sum of those scales.

#### Resting-state session

EEG was recorded during a seven-minute eyes-open resting-state session. Participants were asked to keep their eyes open while looking at the computer screen. Children with TS were not instructed to refrain from ticcing during the resting-state session.

#### EEG recordings

EEG was continuously recorded during the resting-state session. EEG was recorded at 250 Hz with a 128-channel HydroCel Geodesic Sensor Net soaked in potassium chloride solution and a Net Amps 200 amplifier. The Net Station Acquisition software version 4.2.1 (EGI, Inc) was used for data acquisition. Electrode impedance was assessed at or under 40 kΩ before data collection. EEG signals were referenced to the vertex electrode (Cz) and online filtered (0.01–100 Hz bandpass) during acquisition.

### EEG signal treatment

#### EEG preprocessing

Continuous EEG signals were preprocessed with the Maryland Analysis of Developmental EEG pipeline^35^ running on MATLAB 2020a. Detailed preprocessing is described in the Supplement. In short, data were bandpass filtered (1–50 Hz), segmented (two-second epochs), and re-referenced (average reference); bad channels were removed and interpolated; and artifacts were removed with independent component analysis and threshold-based algorithms.

#### Source-based connectivity pipeline

The Brainstorm software was used for source localization.^36^ Brain sources were reconstructed from sensor-level EEG data using weighted minimum-norm estimation. Electrode positions were coregistered to the MNI-ICBM152 template using three reference points (nasion, left, and right preauricular points). A three-layered (scalp, outer skull, inner skull) head model was computed in OpenMEEG^37^ with the symmetric boundary element method. The diagonal of the noise covariance matrix was used in source reconstruction to account for the difference in noise levels across sensors. Cortical parcellation was performed according to the Desikan-Killiany atlas.^38^

Functional connectivity was estimated using the phase-locking value (PLV), which was computed in Brainstorm. The PLV reflects the phase synchrony between two signals, with larger PLV values indicating stronger coupling.^39,40^ The PLV was computed for each two-second epoch with a Hilbert transform in the delta (1–4 Hz), theta (4–8 Hz), alpha (8–13 Hz), beta (13–30 Hz), and gamma (30–50 Hz) frequency bands. The PLV was then averaged across epochs, yielding a single connectivity value for each connection. Functional connectivity was computed between all of the 68 regions of the Desikan-Kiliany atlas.

### Statistical analysis

Continuous demographic and clinical data were analyzed with two-way analyses of variance with the factors ADHD (present/not present) and TS (present/not present). Fisher exact tests comparing the four groups were used for categorical data. For functional connectivity analyses, network-based statistics (NBS)^41^ was used to assess the main effects and interaction of TS and ADHD. Mass univariate testing at each connection was first performed at each connection. Connected graph components were identified among connections exceeding the threshold of α = 0.0005. Permutation testing (5000 permutations) was then performed to compute a *P* value corrected for family-wise error rate. NBS was also used to assess the association between functional connectivity and continuous measures of TS and ADHD symptoms as well as emotional and behavioral problems (YGTSS, SNAP-IV inattention and hyperactivity subscales, DBRS, and CBCL internalizing and externalizing scales) and whether those associations differ according to the TS and ADHD factors, as well as their interaction. For CBCL data, functional connectivity analyses were limited to the internalizing and externalizing scales since scores for individual syndrome scales were not normally distributed. Age was included as a covariate in all functional connectivity analyses. Subnetworks identified through NBS were inspected for extreme values. For subnetworks wherein extreme values (±3 S.D.) were detected, group-specific outliers were removed and the analysis was repeated without those participants.

## Results

### Clinical and demographic data

Clinical and demographic data for each group are presented in Table 1. Groups were mostly equivalent, but the ADHD group was slightly younger and more racially diverse than the others groups. Children with ADHD (ADHD only and TS + ADHD) were more likely than those with TS only to have other co-occurring diagnoses. The TS + ADHD group was the one with the highest rate of medication intake.

### Additive effects of TS and ADHD

**FIGURE 1.**
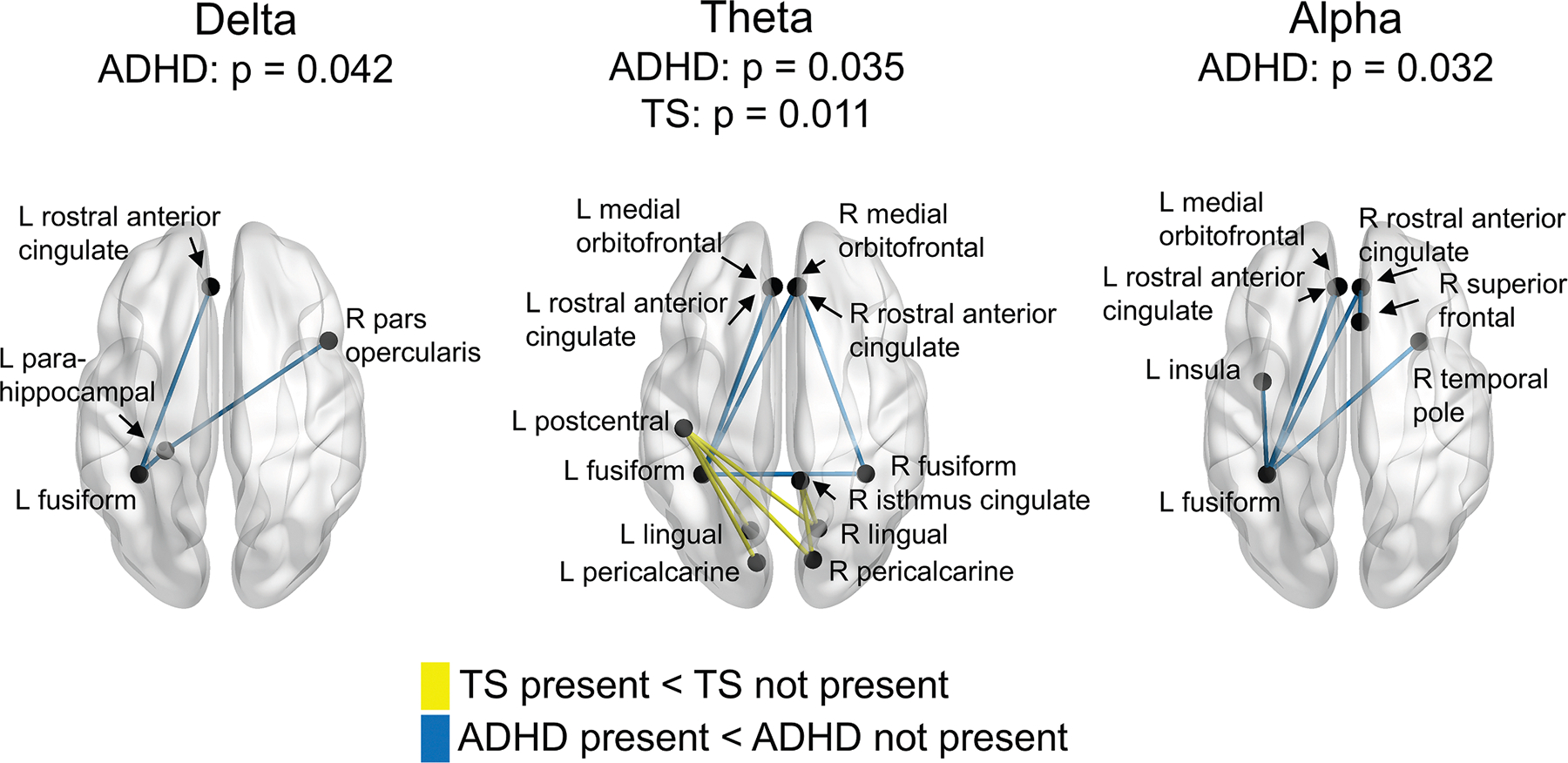
Additive effects of Tourette syndrome (TS) and attention-deficit/hyperactive disorder (ADHD). Analyses revealed significant and distinct subnetworks associated with either TS or ADHD but no interaction between both. For ADHD, three subnetworks with somewhat overlapping connections were found in the delta, theta, and alpha bands. For TS, only one subnetwork in the theta band was significant, with no connection overlapping that of the ADHD subnetwork. The color version of this figure is available in the online edition.

Network-based statistics revealed statistically significant subnetworks of decreased functional connectivity associated with both ADHD and TS in the delta, theta, and alpha frequency bands (Fig 1). In the delta band, ADHD was associated with decreased functional connectivity across several connections (*P* = 0.042). In the theta band, there were distinct and significant subnetworks of decreased functional connectivity associated with ADHD (*P* = 0.035) and TS (*P* = 0.011). In the alpha band, ADHD was associated with a subnetwork of decreased functional connectivity (*P* = 0.032). No interaction between the TS and ADHD factors was found.

### TS/ADHD symptoms and disruptive behaviors

Symptom measures as well as CBCL scores are reported in Table 2. There was no difference between children with TS and those with TS + ADHD regarding YGTSS scores. As expected, children with TS only had less ADHD symptoms than both ADHD groups. However, and even in the absence of a formal ADHD diagnosis, they had more inattention symptoms than typically developing children.

With regard to functional connectivity, network-based statistics revealed no significant main effect or interaction for the YGTSS and the SNAP-IV inattention and hyperactivity subscales, as well as for disruptive behaviors as measured with the DBRS (all *P* values > 0.09).

### Behavioral and emotional problems

Differences across subgroups were observed for all CBCL subscales. Most of these differences were driven by the ADHD factor, but a main effect of TS was observed for the thought problems, internalizing problems, and total problems subscales.

**FIGURE 2.**
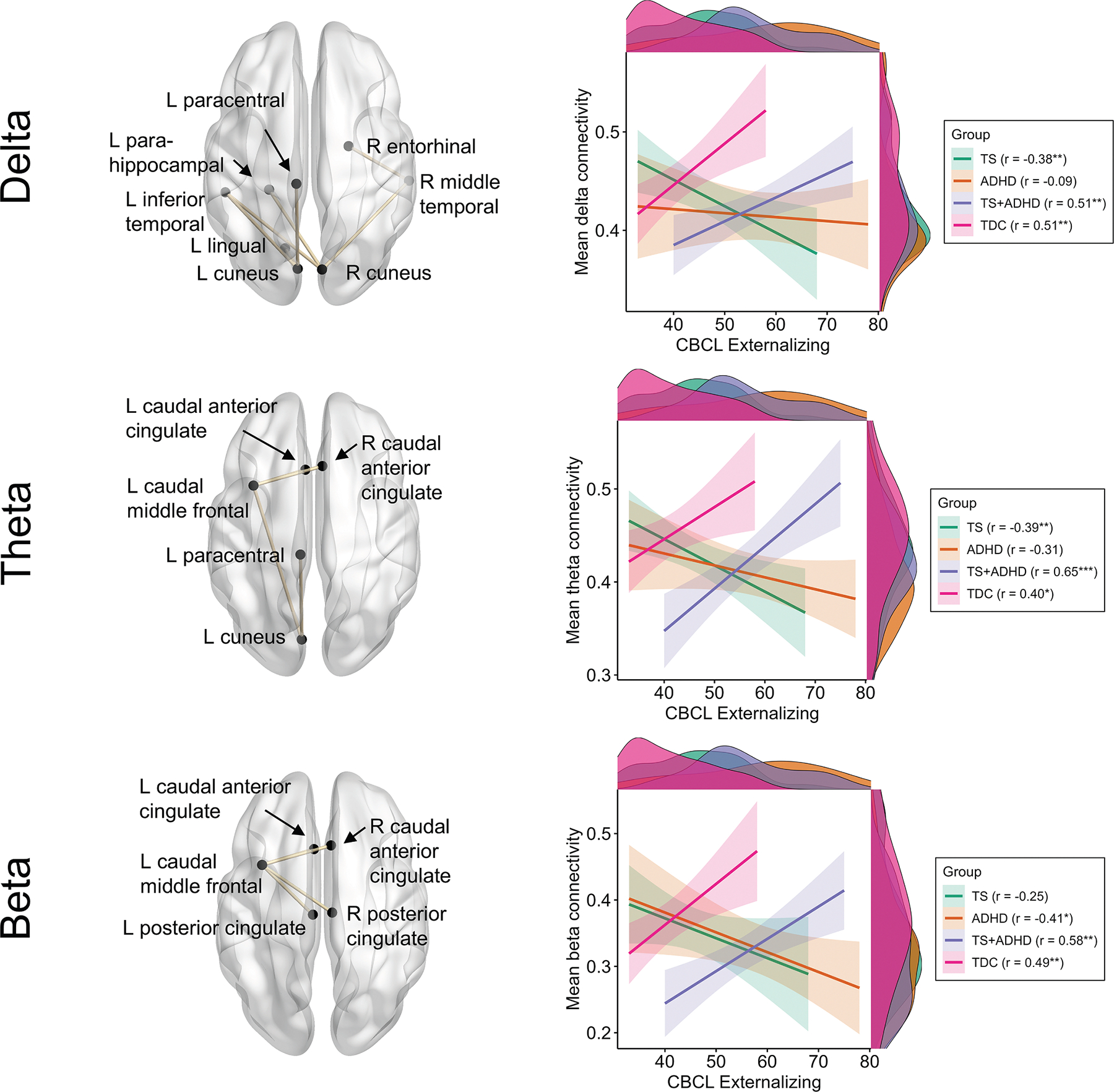
Externalizing problems and functional connectivity. In the delta, theta, and beta frequency bands, there were subnetworks in which we found a Child Behavior Checklist (CBCL) externalizing subscale by attention-deficit/hyperactivity disorder (ADHD) by Tourette syndrome (TS) interaction. Although connections involved in the subnetworks were somewhat distinct (especially for delta), a similar pattern emerged across groups: externalizing problems were significantly associated with increased functional connectivity in those subnetworks for typically developing control subjects and children with TS + ADHD. For children with TS without ADHD and those with ADHD without TS, externalizing problems were either significantly associated with decreased functional connectivity or showed no significant association. Regression lines are presented without data points for the sake of comprehensibility. Scatterplots with data points and regression lines are presented in the Supplement (Figure S1). *P < 0.05, **P < 0.01, ***P < 0.001. The color version of this figure is available in the online edition.

Network-based statistics revealed no main effect of CBCL internalizing and externalizing subscales on functional connectivity (all *P* values > 0.13). No CBCL by ADHD or CBCL by TS interaction was found either (all *P* values = 1.00). However, we found a CBCL externalizing by ADHD by TS interaction in the delta (*P* = 0.019), theta (*P* = 0.037), and beta (*P* = 0.034) frequency bands (Fig 2). No such interaction was observed for the CBCL internalizing scale (all *P* values > 0.05).

## Discussion

The first goal of this study was to assess whether TS and ADHD have additive or interactive effects on functional connectivity. The second goal was to investigate whether functional connectivity was associated with the severity of tics, inattention/hyperactivity symptoms, and disruptive behaviors, as well as with broad indices of emotional/behavioral problems, across groups of children with TS, ADHD, TS + ADHD, and typically developing control subjects.

By contrasting groups on measures of functional connectivity, our analyses revealed additive effects of TS and ADHD. Both conditions were separately associated with reduced functional connectivity across different brain regions and frequency bands. However, no interaction was found. Our results are therefore mostly consistent with previous studies investigating functional connectivity among youth with TS and/or ADHD. For instance, a recent functional connectivity study in children with TS and/or ADHD and typically developing control subjects found no significant difference related to children with TS + ADHD, suggesting the absence of interactive effects.^26^ The study mostly found reduced default mode network connectivity in children with TS relative to typically developing control subjects, as well as reduced frontoparietal network connectivity in children with TS relative to those with ADHD. However, that study did not use a 2 × 2 factorial design, which would allow for testing whether TS and ADHD had additive or interactive effects on functional connectivity. Another study, employing high-density electroencephalography and effective brain connectivity, found additive effects for both ADHD and TS.^24^ The ADHD factor was associated with decreased effective connectivity between several cortical regions (mostly in posterior regions), whereas the TS factor was associated with increased connectivity from the left postcentral to the right precuneus and reduced connectivity from the left occipital cortex to the right precuneus.

Among children with ADHD, our analyses revealed reduced functional connectivity in the three slowest frequency bands: delta, theta, and alpha. Two regions, the left fusiform gyrus and the left rostral anterior cingulate cortex, were involved in subnetworks of decreased functional connectivity across the three frequency bands. In the theta and alpha frequency bands, decreased functional connectivity between the fusiform gyrus and the medial orbitofrontal cortex was found as well. The rostral anterior cingulate cortex shares important connections with the orbitofrontal cortex,^42^ and both play an important role in emotion processing and regulation.^43^ Here, reduced functional connectivity may reflect patterns inherent to ADHD, as several previous studies reported decreased functional connectivity^44^ and increased cortical thinning^45,46^ in those areas among individuals with ADHD. Our findings thus suggest disruptions in functional connectivity among distributed cortical areas in children with ADHD.

In TS, reduced functional connectivity was only found in the theta band. The subnetwork of decreased functional connectivity included connections between the left postcentral gyrus and the bilateral lingual gyri and pericalcarine cortices, as well as right-hemisphere connections between the isthmus of the cingulate and the lingual gyrus and pericalcarine cortex. These findings are consistent with prior research investigating structural connectivity in TS, which has reported reduced graph theoretical metrics of structural connectivity in several of these regions.^47^ Furthermore, regions where we found reduced functional connectivity in children with TS are mostly included in the sensorimotor (postcentral gyrus) and visual (lingual gyri and pericalcarine cortices) networks. The postcentral gyrus, which corresponds to the primary somatosensory cortex, plays an important role in the integration of somatosensory stimuli and is particularly relevant in TS. Reduced activity in this region has notably been associated with deficient sensorimotor gating in children with TS.^48^ Furthermore, gray matter volumes of the somatosensory cortex have been associated with premonitory urges.^49^ In addition, reduced connectivity between sensorimotor and visual networks may reflect difficulties in integrating sensory and visual information. For instance, children with TS showed worse performance during an eyes-open postural task, suggesting difficulties with the integration of visual information necessary for dynamic postural control.^50^ Additionally, prior research has shown that individuals with TS depend more on visual feedback when performing movements, suggesting altered proprioceptive processing.^51^ Such difficulties with integration of visual information may thus result from reduced functional connectivity between occipitals and somatosensory regions.

In the current study, functional connectivity was measured in five frequency bands. Assessing the inter-regional synchronization of neural oscillations across several frequency bands is important given that network organization properties have been found to fluctuate in a frequency-specific way.^27^ Also, low frequencies tend to favor long-range connectivity, whereas high frequencies tend to favor short-range connectivity.^52,53^ Thus, resting-state networks may have distinct functional significance across frequencies. For instance, theta oscillations, which are relatively slow, are associated with top-down cognitive control.^54^ Additionally, as a slow frequency, theta allows long-range communication of distributed brain regions involved in cognitive control.^55^ Thus, reduced theta functional connectivity may potentially reflect diminished integration across cortical regions leading to difficulties with cognitive control in TS^56^ and ADHD.^57^ Also, although the methods used by Jurgiel et al.^24^ favored broadband connectivity effects, most of the altered connections they reported in children with ADHD spanned frequencies included in the theta band. Thus, it seems that resting-state theta band dysconnectivity may be an important feature in children with ADHD. The reduced functional connectivity associated with the ADHD factor in the delta and alpha bands may reflect broad dysregulation across multiple frequency bands. Delta and alpha activity have, respectively, been associated with motivational^58,59^ and attentional processes,^60–62^ which are known to be impacted in ADHD.^63,64^ The absence of TS-related effects in the other frequency bands is also consistent with the notion that TS is associated with less-widespread impairment compared with ADHD, both in terms of emotional difficulties, as reflected in the CBCL scores from the current study, and cognitive impairments, as consistently reported in the literature.^56,57^

The comparison of functional connectivity across children with and without TS and/or ADHD revealed additive effects, whereas a different pattern emerged when looking at associations with emotional and behavioral functioning. We found no main effect of either internalizing or externalizing problems, or ADHD or TS. However, our analyses revealed TS by ADHD by externalizing problems interactions across three frequency bands. In the delta, theta, and beta bands, enhanced functional connectivity among distributed cortical regions was positively associated with externalizing symptoms in children with TS + ADHD and TDC. In children with TS without ADHD, externalizing symptoms were significantly and negatively associated with functional connectivity in the delta and theta bands. This was only the case in the beta band for children with ADHD without TS. Thus, although children with ADHD without TS and those with TS + ADHD show enhanced externalizing symptoms compared with the two other groups, it seems that the neurobiological correlates of such symptoms differ across diagnostic groups. It is possible that the behaviors that parents rated as externalizing differ for children with different profiles of co-occurring TS and ADHD resulting in different neurobiological correlates for different behaviors. Those results are consistent with and also extend the stepwise model proposed by Banaschewski et al.^13^ For basic neurobiological aspects, such as functional connections differing across TS and ADHD, we found additive effects. However, when assessing the functional connections associated with externalizing symptoms, we found interactive effects. Such dissociation is not new: prior research has shown that TS and ADHD may interact for high cognitive demands while being additive for lower cognitive load.^19^ Thus, whether TS and ADHD are additive or interactive may depend on the level of investigation.^13,14,20^

The results of this study must be interpreted in the context of some limitations. Although source-EEG functional connectivity allows assessment of the synchronization of neural oscillations across distinct frequency bands, its spatial accuracy is not as precise as that of functional magnetic resonance imaging. Replication of our findings with other techniques such as functional magnetic resonance imaging or functional near-infrared spectroscopy may be useful here. Another limitation consists in the categorization of ADHD in children with TS. In the current study, children with TS without ADHD had more parent-reported inattentive symptoms than typically developing children, even in the absence of a formal ADHD diagnosis. Some subclinical ADHD symptoms may be inherent to TS and could complicate the assessment of additive and interactive effects of TS and ADHD. Also, other confounding factors may have influenced our results. For instance, our analyses did not consider medication intake or the number of years since tic onset. These are important aspects of TS that should be considered in future studies. Finally, our clinical groups were predominantly male, especially the TS and TS + ADHD groups. Future research should make it a priority to recruit more girls to have more representative samples and a better understanding of TS in girls.

In conclusion, the current study revealed additive effects of TS and ADHD on source-EEG functional connectivity. However, when assessing associations between functional connectivity and measures of internalizing and externalizing symptoms, our analyses revealed interaction effects between TS and ADHD. These findings are consistent with proposals that TS and ADHD may be additive for basic neurobiological aspects but may interact for more complex processes. Future work considering the complexity and variety of ADHD symptoms in TS is needed to help untangle those two conditions.

## Supplementary Material

Supplement

Supplementary data

Supplementary data related to this article can be found at https://doi.org/10.1016/j.pediatrneurol.2025.08.001.

## Figures and Tables

**TABLE 1.** Demographic and Clinical Characteristics of Study Participants

Characteristics	TS (n = 51)	ADHD (n = 24)	TS + ADHD (n = 29)	TDC (n = 33)	TS Main Effect (ANOVA)	ADHD Main Effect (ANOVA)	TS by ADHD Interaction (ANOVA)	Categorical Variables (Fisher Exact Test)	Group Comparison

Age in years, mean (S.D.)	11.3 (2.0)	10.0 (2.0)	11.3 (1.7)	11.4 (1.6)	n.s.	F_1,133_ = 4.51, *P* = 0.035	F_1,133_ = 4.32, *P* = 0.039	-	TS/TS + ADHD/TDC > ADHD
Sex (% male)	88.2%	79.2%	86.2%	69.7%	-	-	-	n.s.	-
Handedness (% right handed)*	80.0%	86.3%	85.7%	90.9%	-	-	-	n.s.	-
Race, number (%)^†^					-	-	-	*P* = 0.037	TS/TS + ADHD/TDC > ADHD
White	38 (84.4%)	13 (54.2%)	25 (86.2%)	29 (87.8%)	-	-	-	-	-
Black	4 (7.8%)	3 (12.5%)	1 (3.4%)	1 (3.0%)	-	-	-	-	-
American Indian	0 (0%)	1 (4.2%)	0 (0%)	0 (0%)	-	-	-	-	-
Asian	3 (5.9%)	0 (0%)	1 (3.4%)	3 (9.1%)	-	-	-	-	-
Biracial	0 (0%)	4 (16.7%)	0 (0%)	0 (0%)	-	-	-	-	-
Missing/not reported	6 (11.8%)	3 (12.5%)	1 (3.4%)	0 (0%)	-	-	-	-	-
Ethnicity (% Hispanic)	1 (2.2%)	3 (13.6%)	3 (10.7%)	1 (3.0%)	-	-	-	n.s.	-
Co-occurring diagnoses, number (%)									
Any condition (other than ADHD)	10 (19.6%)	11 (45.8%)	15 (51.7%)	0 (0%)	-	-	-	*P <* 0.001	ADHD/TS + ADHD > TS > TDC
OCD	5 (9.8%)	0 (0%)	6 (20.7%)	0 (0%)	-	-	-	-	
ODD	2 (3.9%)	5 (20.8%)	8 (27.6%)	0 (0%)	-	-	-	-	
Conduct disorder	0 (0%)	6 (25%)	0 (0%)	0 (0%)	-	-	-	-	
Any anxiety disorder	6 (11.8%)	1 (4.2%)	5 (17.2%)	0 (0%)	-	-	-	-	
Medication status, number (%)									
On psychotropic medication	15 (29.4%)	6 (25%)	17 (58.6%)	1 (3.4%)	-	-	-	*P* < 0.001	TS/ADHD/TS + ADHD > TDC, TS + ADHD > ADHD
Stimulants^‡^	0 (0%)	6 (25%)	5 (17.2%)	0 (0%)	-	-	-	-	-
α-Agonists^§^	10 (19.6%)	1 (4.2%)	9 (31.0%)	0 (0%)	-	-	-	-	-
Atomoxetine	0 (0%)	0 (0%)	3 (10.3%)	0 (0%)	-	-	-	-	-
Antipsychotics^||^	3 (5.9%)	1 (4.2%)	8 (27.6%)	0 (0%)	-	-	-	-	-
SSRIs^¶^	3 (5.9%)	0 (0%)	5 (17.2%)	1 (3.4%)	-	-	-	-	-
Other^#^	0 (0%)	0 (0%)	2 (6.9%)	0 (0%)	-	-	-	-	-

Abbreviations:

ADHD = Attention-deficit/hyperactivity disorder

ANOVA = Analysis of variance

n.s. = non-significant

OCD = Obsessive-compulsive disorder

ODD = Oppositional defiant disorder

SSRI = Selective serotonin reuptake inhibitors

TDC = Typically developing control subjects

TS = Tourette syndrome

* Data were binarized as right-handed/other for statistical analysis.

† Data were binarized as white/other for statistical analysis.

‡ Stimulant medications included methylphenidate (n = 6), lisdexamfetamine (n = 3), dexmethylphenidate (n = 1), and dextroamphetamine (n = 1).

§ α-Agonists included guanfacine (n = 17) and clonidine (n = 3).

|| Antipsychotics included risperidone (n = 7), haloperidol (n = 2), aripiprazole (n = 2), and quetiapine (n = 1).

¶ SSRIs included citalopram (n = 2), fluvoxamine (n = 2), sertraline (n = 2), fluoxetine (n = 2), and escitalopram (n = 1).

# Other medications included benztropine (n = 1) and gabapentin (n = 1).

**TABLE 2.** Scores on Clinician-Administered and Parent-Reported Clinical Scales

Scores	TS (n = 51)	ADHD (n = 24)	TS + ADHD (n = 29)	TDC (n = 33)	TS Main Effect (ANOVA)	ADHD Main Effect (ANOVA)	TS by ADHD Interaction (ANOVA)	Group Comparison

Clinical scores, mean (S.D.)								
YGTSS total tic score	24.0 (7.1)	-	25.1 (8.5)	-	n.s.	n.s.	n.s.	-
SNAP-IV total	9.5 (6.9)	28.8 (11.9)	26.3 (10.9)	4.5 (4.4)	n.s.	F_1,128_ = 180.25, *P* < 0.001	F (1,128) = 5.95, *P* = 0.016	ADHD/TS + ADHD > TS > TDC
SNAP-IV inattentive	5.0 (3.9)	13.6 (5.6)	12.6 (5.1)	2.1 (2.3)	n.s.	F_1,126_ = 159.71, *P* < 0.001	F (1,126) = 6.81, *P* = 0.010	ADHD/TS + ADHD > TS > TDC
SNAP-IV hyperactive	4.5 (3.7)	15.3 (6.9)	13.7 (6.4)	2.6 (2.7)	n.s.	F_1,126_ = 151.88, *P* < 0.001	n.s.	ADHD/TS + ADHD > TS/TDC
DBRS total	4.6 (4.5)	9.6 (6.0)	8.5 (4.7)	2.9 (3.5)	n.s.	F_1,124_ = 39.88, *P* < 0.001	n.s.	ADHD/TS + ADHD > TS/TDC
CBCL								
Anxious/depressed	55.4 (7.2)	58.4 (9.7)	58.3 (7.4)	51.2 (4.6)	n.s.	F_1,124_ = 15.10, *P* < 0.001	n.s.	TS/ADHD/TS + ADHD > TDC
Withdrawn/depressed	54.9 (7.4)	57.4 (8.1)	55.2 (6.1)	51.7 (2.9)	n.s.	F_1,124_ = 6.87, *P* = 0.010	F_1,124_ = 5.39, *P* = 0.022	TS/ADHD/TS + ADHD > TDC
Somatic complaints	57.3 (7.8)	58.2 (8.9)	55.8 (6.9)	51.3 (2.9)	n.s.	F_1,124_ = 4.66, *P* = 0.033	F_1,124_ = 11.28, *P* = 0.001	TS/ADHD/TS + ADHD > TDC
Social problems	52.8 (8.6)	58.1 (8.4)	59.1 (8.2)	51.1 (1.7)	n.s.	F_1,124_ = 24.41, *P* < 0.001	n.s.	ADHD/TS + ADHD > TS/TDC
Thought problems	59.6 (8.6)	59.5 (9.5)	63.1 (8.1)	51.2 (3.4)	F_1,124_ = 18.82, *P* < 0.001	F_1,124_ = 18.06, *P* < 0.001	n.s.	TS/ADHD/TS + ADHD > TDC
Attention problems	53.2 (5.0)	63.2 (8.9)	61.6 (9.6)	51.3 (2.1)	n.s.	F_1,124_ = 72.67, *P* < 0.001	n.s.	ADHD/TS + ADHD > TS > TDC
Rule-breaking behavior	51.8 (3.0)	57.5 (7.0)	55.4 (6.8)	51.0 (1.7)	n.s.	F_1,124_ = 33.46, *P* < 0.001	n.s.	ADHD/TS + ADHD > TS/TDC
Aggressive behavior	52.8 (4.6)	62.0 (11.6)	58.1 (8.5)	51.1. (2.7)	n.s.	F_1,124_ = 41.19, *P* < 0.001	F (1,124) = 4.73, *P* = 0.032	ADHD/TS + ADHD > TS > TDC
Internalizing problems	52.5 (11.7)	55.9 (12.9)	55.1 (10.5)	43.5 (7.6)	F_1,124_ = 4.41, *P* = 0.038	F_1,124_ = 15.02, *P* < 0.001	F_1,124_ = 6.43, *P* = 0.012	TS/ADHD/TS + ADHD > TDC
Externalizing problems	46.3 (8.9)	57.7 (13.0)	55.7 (9.7)	41.6 (8.0)	n.s.	F_1,124_ = 52.49, *P* < 0.001	n.s.	ADHD/TS + ADHD > TS > TDC
Total problems	49.7 (10.0)	59.1 (12.7)	58.7 (8.3)	40.0 (8.6)	F_1,124_ = 6.96, *P* = 0.009	F_1,124_ = 62.57, *P* < 0.001	F_1,124_ = 8.23, *P* = 0.005	ADHD/TS + ADHD > TS > TDC

Abbreviations:

ADHD = Attention-deficit/hyperactivity disorder

ANOVA = Analysis of variance

CBCL = Child Behavior Checklist

DBRS = Disruptive Behavior Rating Scale

n.s. = non-significant

SNAP-IV = Swanson, Nolan and Pelham Questionnaire for ADHD

TDC = Typically developing control subjects

TS = Tourette syndrome

YGTSS = Yale Global Tic Severity Scale
