# Supplementary material for: Electroencephalographic Functional Connectivity Patterns in Children With Tourette Syndrome and Attention-Deficit/Hyperactivity Disorder
Source: Pediatr Neurol. Author manuscript; Available in PMC 2026 Mar 16. (PMC12991204; doi:10.1016/j.pediatrneurol.2025.08.001)
Supplement: Supplement [file NIHMS2148211-supplement-Supplement.docx]

**Electroencephalographic Functional Connectivity Patterns in Children With Tourette Syndrome and Attention-Deficit/Hyperactivity Disorder**

Simon Morand-Beaulieu, Julia Zhong, Karim Ibrahim, Michael J. Crowley, Heidi Grantz, James F. Leckman, Denis G. Sukhodolsky

***Supplement***

**Supplementary methods**

*EEG preprocessing*

The Maryland Analysis of Developmental EEG (MADE) pipeline is a Matlab script for preprocessing infants’ and children’s EEG data (Debnath et al., 2020). It uses EEGLAB’s (Delorme and Makeig, 2004) data structure and functions. It involves multiple and sequential steps, which are described below.

1. Filtering

Data were offline filtered with EEGLAB’s firfilt plugin, which uses a Hamming window finite impulse response (FIR) filter. Data were first filtered with a 1 Hz high-pass filter (0.3 Hz transition width), and then with a 50 Hz low-pass filter (10 Hz transition width). The stopband attenuation was 53 dB.

2. Identification and removal of bad channels

Bad channels were identified with the channel_properties function of the FASTER plugin (Nolan et al., 2010). Bad channels were identified according to three indicators: Hurst exponent, correlation with other channels, and channel variance. Bad channels were removed at this point of the preprocessing.

3. Artifact removal using independent component analysis (ICA)

Non-neural artifacts such as blinks, saccades, and muscle artifacts were removed with an ICA-based method. Given that ICA performs better on data filtered with higher high-pass filter, a copy of the EEG recordings was made. This copy was high-pass filtered at 1 Hz and epoched into 1-second segments. Any 1-second segment with very low/high amplitude epochs (± 1000 μV) and excessive EMG activity (exceeding -30 and 100 dB between 20-40 Hz) was identified as bad. Bad segments were removed from the copied dataset. Also, any channel in which more than 20% of 1-second segments were bad was removed from both the original and copied dataset. ICA was the performed on the copy of the dataset using EEGLAB’s runica function. Then, ICA weights were transferred to the original dataset. Independent components representing artifacts were identified with the adjusted-ADJUST algorithm (Leach et al., 2020), which is version of EEGLAB’s ADJUST plugin (Mognon et al., 2011) adapted for EEG recordings in children and adolescents (Leach et al., 2020). Artifacted independent components were removed from the original dataset.

4. Segmentation

Continuous data was then epoched into 2-seconds segments with 50% overlap.

5. Threshold-based rejection of residual artifacts

A threshold-based artifact rejection was used to deal with residual artifacts following ICA. First, epochs were removed if voltage exceeded ±100 μV in a frontal channel close to the eyes (1, 8, 14, 21, 25, 32). Then, epochs in which more than 10% of channels had voltage exceeding ±100 μV were removed. Finally, for the remaining epochs, channels with voltage exceeding ±100 μV were interpolated at the epoch level using a spherical spline procedure.

6. Channel interpolation

Channels that were removed prior to performing ICA were then interpolated using a spherical spline procedure.

7. Re-referencing

Finally, electrodes were re-referenced to the average of all electrodes (average reference).

**Supplementary results**


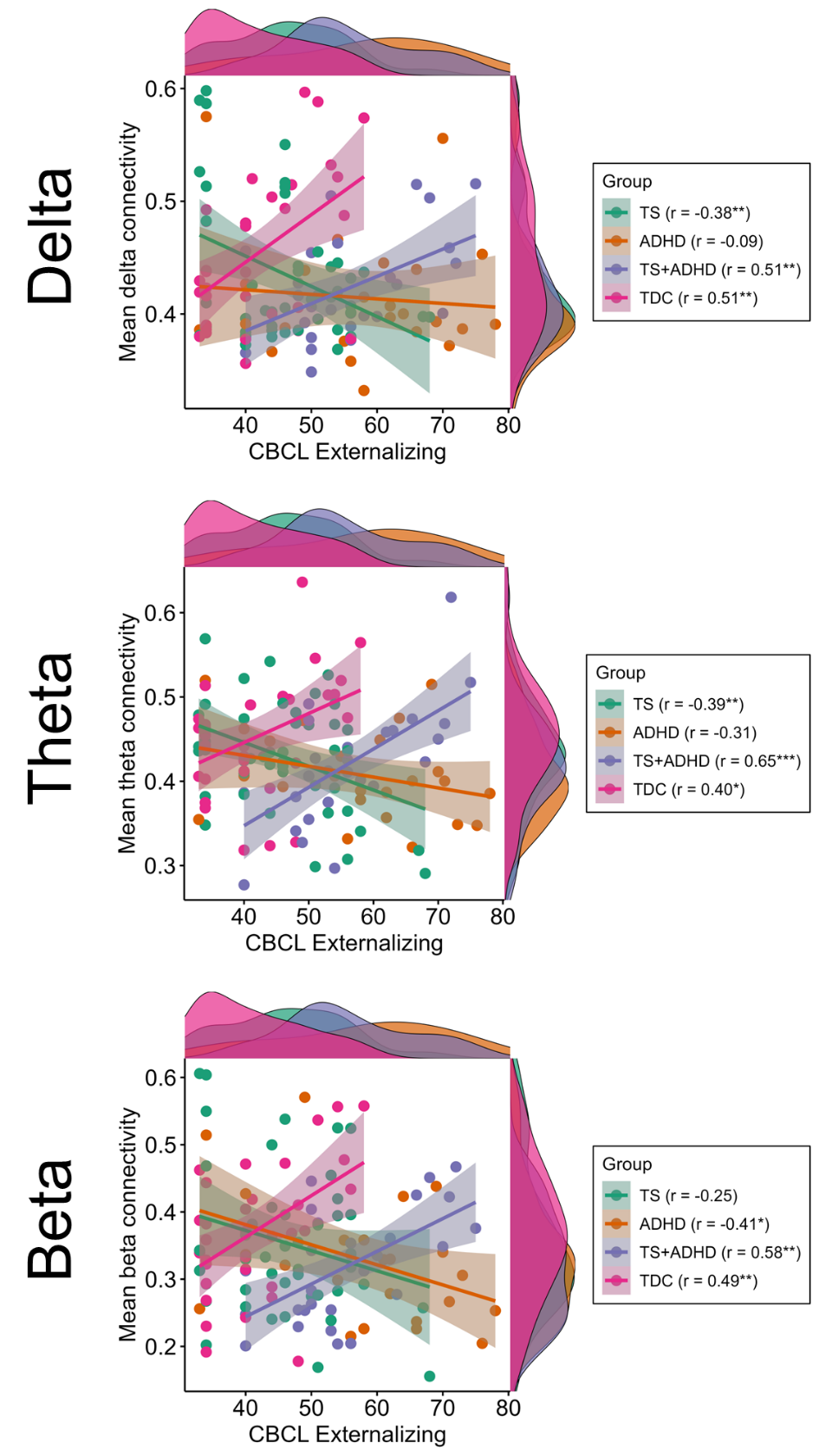


**Figure S1: Scatterplots of the associations between externalizing problems and functional connectivity.** In Figure 2, regression lines were presented without data points for the sake of comprehensibility. Here are depicted the scatterplots showing the association between externalizing problems and functional connectivity in the delta, theta, and beta-frequency subnetworks were interactions were found.

**References**

Debnath, R., Buzzell, G.A., Morales, S., Bowers, M.E., Leach, S.C., Fox, N.A., 2020. The Maryland analysis of developmental EEG (MADE) pipeline. Psychophysiology 57(6), e13580.

Delorme, A., Makeig, S., 2004. EEGLAB: an open source toolbox for analysis of single-trial EEG dynamics including independent component analysis. Journal of Neuroscience Methods 134(1), 9-21.

Leach, S.C., Morales, S., Bowers, M.E., Buzzell, G.A., Debnath, R., Beall, D., Fox, N.A., 2020. Adjusting ADJUST: Optimizing the ADJUST algorithm for pediatric data using geodesic nets. Psychophysiology 57(8), e13566.

Mognon, A., Jovicich, J., Bruzzone, L., Buiatti, M., 2011. ADJUST: An automatic EEG artifact detector based on the joint use of spatial and temporal features. Psychophysiology 48(2), 229-240.

Nolan, H., Whelan, R., Reilly, R.B., 2010. FASTER: Fully Automated Statistical Thresholding for EEG artifact Rejection. Journal of Neuroscience Methods 192(1), 152-162.
